# Molecular Mechanisms in Autoimmune Thyroid Disease

**DOI:** 10.3390/cells12060918

**Published:** 2023-03-16

**Authors:** Hernando Vargas-Uricoechea

**Affiliations:** Metabolic Diseases Study Group, Department of Internal Medicine, Universidad del Cauca, Carrera 6 Nº 13N-50, Popayán 190001, Colombia; hernandovargasuricoechea@gmail.com; Tel.: +57-3163121411; Fax: +57-8234712

**Keywords:** thyroid, autoimmunity, Graves-Basedow, Hashimoto, pathogenesis, genetic, environmental, epigenetic

## Abstract

The most common cause of acquired thyroid dysfunction is autoimmune thyroid disease, which is an organ-specific autoimmune disease with two presentation phenotypes: hyperthyroidism (Graves-Basedow disease) and hypothyroidism (Hashimoto’s thyroiditis). Hashimoto’s thyroiditis is distinguished by the presence of autoantibodies against thyroid peroxidase and thyroglobulin. Meanwhile, autoantibodies against the TSH receptor have been found in Graves-Basedow disease. Numerous susceptibility genes, as well as epigenetic and environmental factors, contribute to the pathogenesis of both diseases. This review summarizes the most common genetic, epigenetic, and environmental mechanisms involved in autoimmune thyroid disease.

## 1. Introduction

Autoimmune diseases (ADs) are a heterogeneous group of more than 100 pathological conditions that are characterized by an alteration in the regulation of inflammatory processes against one or multiple autoantigens [1,2].

The prevalence of ADs is variable. It is estimated that 3–10% of the general population has at least one AD, and among them, 5–10% have another AD. In addition, 80% of all diagnosed patients are women [3,4].

ADs are usually classified as organ-specific (OS) or non-organ-specific (NOS), depending on whether they affect one organ or several; in NOS ADs, the autoimmune activity is systemic (as in systemic lupus erythematosus). In OS ADs, the immune response is directed toward single-organ antigens. The most common OS AD is autoimmune thyroid disease (AITD) [5,6,7,8].

The AITD spectrum includes Graves–Basedow disease (GBD) and Hashimoto’s thyroiditis (HT), with two extremes of clinical presentation: hyperthyroidism (in the case of GBD) and hypothyroidism (in the case of HT). However, cases can be identified in which, in the presence of thyroid autoimmunity, no clinical or biochemical manifestations of hypothyroidism or hyperthyroidism are identified.

Additionally, some subjects with HT may progress to GBD (or vice versa), and it is possible to identify individuals with simultaneous manifestations of both GBD and HT [9,10].

In fact, some individuals with biochemical findings of thyroid autoimmunity, typical of GBD (with the presence of thyroid ophthalmopathy), may present hypothyroidism (even requiring levothyroxine replacement). Similarly, some individuals with biochemical evidence of autoimmunity, typical of HT, may present with long-standing hyperthyroidism (with associated ophthalmopathy and dermopathy).

In GBD, there is a loss of immune tolerance, with the infiltration of T lymphocytes (TLs) in the thyroid and the activation of B lymphocytes (BLs), as well as an increase in the synthesis and secretion of autoantibodies directed against the TSH receptor (TSHR). Consequently, the interaction between the TSHR and its specific autoantibody (TRAb) causes an immune response that results in goiter, hyperthyroidism, ophthalmopathy, and dermopathy [11].

In HT, there is a cellular immune response with a high inflammatory load and apoptosis, which causes tissue destruction and thyroid dysfunction. HT also shares humoral mechanisms with GBD, with the presence of autoantibodies (Abs) against thyroid peroxidase (TPO) and thyroglobulin (Tg) [12].

It is generally accepted that AITD is the product of multiple environmental factors that act based on genetic susceptibility, together with some epigenetic mechanisms. However, the molecular mechanism by which immune dysfunction leads to the destruction of thyroid tissue remains largely unexplained [13,14,15,16,17].

The objective of this review is to describe the main molecular mechanisms that lead to AITD.

## 2. Methods (Search Strategy)

**Figure 1:**
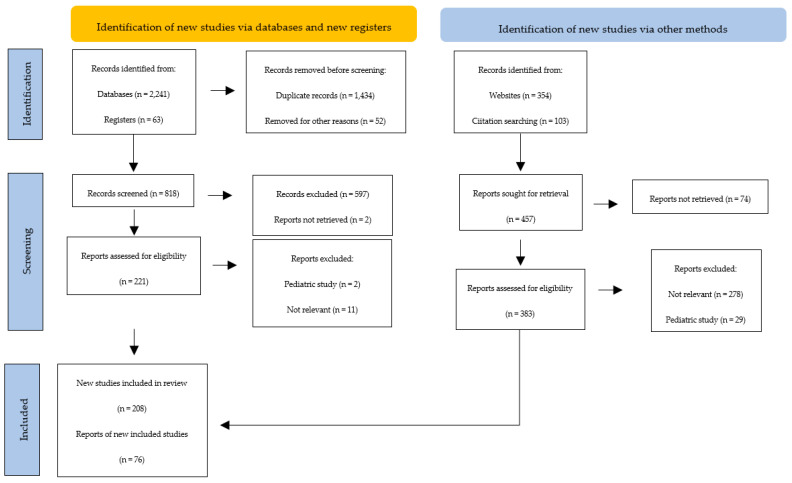
PRISMA flow diagram. Method for the selection of articles for this review.

A detailed search was carried out in the following databases: PubMed/MEDLINE, EMBASE, Scopus, BIOSIS, Web of Science, and Cochrane Library. This search was conducted for articles published with no date limit and using the following keywords: autoimmune thyroiditis, Hashimoto thyroiditis, Graves–Basedow disease, and autoimmune thyroid disease. Only articles written in English were taken into account (Figure 1).

## 3. Animal Models of AITD

The experimental models of ADs in animals are of two types: *spontaneous*, in which animals with or without genetic modifications develop the disease spontaneously, and *induced*, in which the outcome is developed artificially. In animal models of induced AITD, the strategy is based on the use of crude thyroid extracts, purified Tg or TPO, and selected ectodomains. Of these models, the best studied use nonobese diabetic (NOD) mice, which highlights the fact that NOD mice can develop different experimental ADs (including AITD) [18,19,20,21].

**Figure 2:**
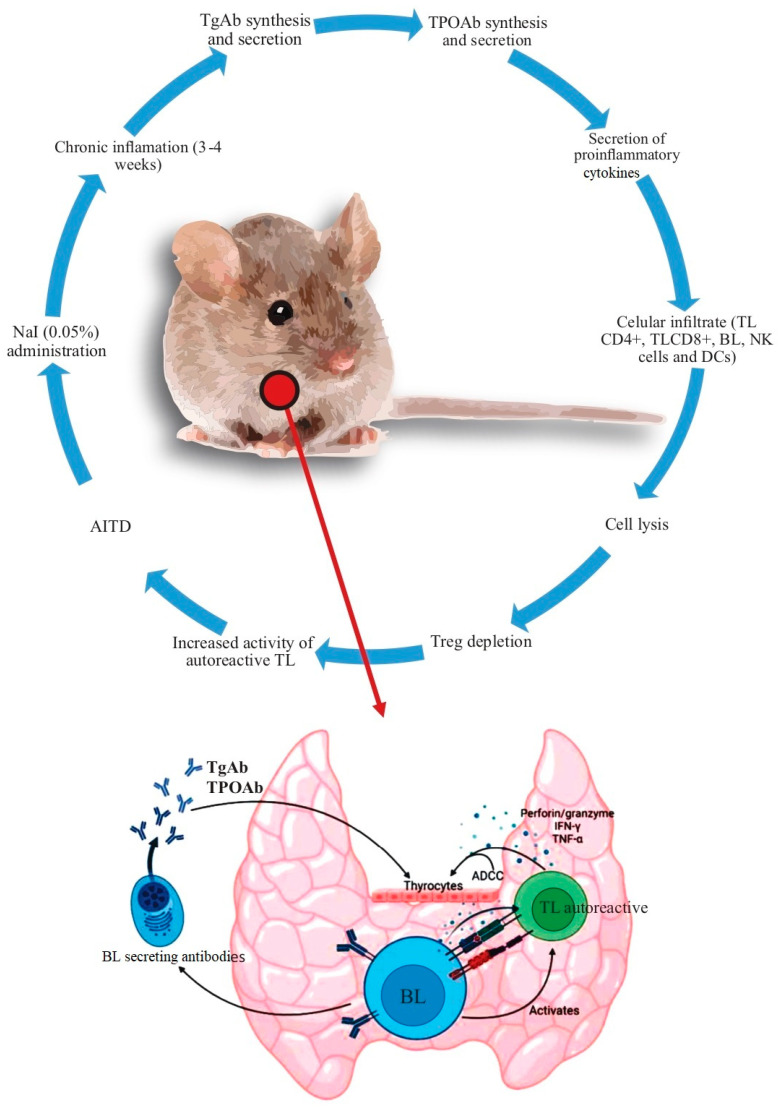
Summary of the mechanisms leading to AITD in the non–obese diabetic (NOD) mice. After the administration of 0.05% NaI in the drinking water, there were findings of chronic inflammation in the following 3–4 weeks. Subsequently, the synthesis of autoantibodies increases (initially against Tg and later against TPO). At the same time, there is an increase in the secretion of proinflammatory cytokines and cell infiltration [mediated initially by TL (CD4+) and then by TL (CD8+), macrophages, and, finally, by BL, although other cells, such as TL, NK, and DCs, among others, also participate in this process]. Additionally, there is an increased production of ADCC, perforin/granzyme, IFN–γ, and TNF–α. TReg depletion increases the severity of the immune response, with loss of immune tolerance and the development of AITD. Abbreviations: ADCC: antibody dependent cell cytotoxicity, BL: B lymphocyte, DCs: dendritic cells, NK: natural killer, Tg: thyroglobulin, TL: T lymphocyte, TPO: thyroid peroxidase, Treg: regulatory T lymphocyte.

Animal models of AITD have greatly aided our understanding of its pathogenesis. The findings found with NOD mice concerning AITD are summarized in Table 1 and Figure 2.

## 4. Genetic Factors

AITD is considered a familial disease, as a family history is found among GBD patients in >60% of cases, and accumulations of GBD and HT have also been demonstrated (in relatives of the index case). Similarly, in monozygotic (MZ) twins, it has been found that if GBD is present in one twin, then HT can develop in the other (which suggests that the genetic factors that predispose to one of the two diseases can potentially increase the risk of the other) [43,44,45,46,47,48,49,50].

There is also a greater concordance of GBD in MZ twins; for example, the concordances are 35% and 3% in MZ and dizygotic (DZ) twins, respectively. In HT, concordance rates of 55% and 0% have been found in MZ and DZ twins, respectively, together with positivity concordance rates of 59% and 23% for TgAbs and 47% and 29% for TPOAbs in MZ and DZ twins, respectively [46,51,52,53,54].

The familial clustering of AITD (sibling risk ratio) has been estimated to be from 5.9 to >10 (a value of >5.0 is considered significant) [55,56,57,58].

Moreover, a high recurrence has been demonstrated in the first- and second-degree relatives of patients with AITD. Among the GBD patients, 6.1% of the first-degree relatives had GBD, and among the HT patients, 4.9% of the first-degree relatives were affected. A Mendelian dominant inheritance pattern for the tendency to develop thyroid autoantibodies has been suggested [58,59,60,61,62].

The identification of the susceptibility genes for AITD has allowed a better understanding of the triggering mechanisms, which suggests that the presence of these genes (along with interactions with some environmental and epigenetic factors) exacerbates the risk of AITD [58,63,64].

The susceptibility genes for AITD can be classified as those that are related to the immune system (*HLA-DR3*, *PTPN22*, *CD40*, *FOXP3*, and *CTLA-4*, among others) and those that are related to the thyroid, such as the genes that encode for the synthesis of the major thyroid autoantigens (*Tg*, *TPO*, and the *TSHR*) [15,58,65,66].

### 4.1. Susceptibility Genes for AITD Associated with Immune System

#### 4.1.1. *HLA-DR3*

The human leukocyte antigen (HLA) system is a cluster of gene complexes encoding the major histocompatibility complex (MHC) proteins. The cell-mediated adaptive immune response is regulated by the HLA, and its primary function is to present endogenous and exogenous antigens to TLs for recognition and response. The HLA molecules that present antigens to TLs are divided into two main classes: HLA class I (HLA-I) and HLA class II (HLA-II). HLA-I molecules play an essential role in the immune defense against intracellular pathogens, whereas HLA-II molecules are predominantly involved in displaying peptides from extracellular pathogens [67].

HLA polymorphisms (SNPs) determine the HLA diversity and its association with various diseases, as they can determine the specificity of the binding to a specific antigen and the initiation of the immune response, as well as influence the differentiation of TLs in the thymus, modifying the regulation of the response [67,68].

The HLA also controls cytokine synthesis and secretion; therefore, certain HLA susceptibility alleles could lead to AITD (probably by preferentially regulating the Th2 (in GBD) and Th1 (in HT) pathways). HLA-A*68 and HLA-B*08 have also been found to confer GBD susceptibility, while HLA-A*33, HLA-DQB1*0201, and HLA-DQA1*0201 appear to have protective functions [69].

Recently, a significant association was demonstrated between Grave’s ophthalmopathy and HLA-A*01:01, HLA-A*32:01, HLA-B*37:01, HLA-B*39:01, HLA-B*42:01, HLA-C*08:02, HLA-C*03:02, HLA-DRB1*03:01, HLA-DRB1*14:01, and HLA-DQB1*02:01, while HLA-C*04:01, HLA-C*03:04, HLA-C*07:02, and HLA-DRB1*15:02 were shown to be protective alleles [70].

In another study, it was found that the SNPs rs3177928 and rs7197 were correlated with AITD and GBD (compared with the healthy control group), but not with HT. However, rs3177928 and rs7197 were correlated with AITD and HT in the allele model, dominant model, and overdominant model before and after gender and age adjustment, but not with HT [71].

Table 2 summarizes the most recent studies on HLA and AITD.

Currently, of all the HLA subtypes, *HLA-DR3* is the most strongly associated with AITD, especially since 40–50% of GBD patients harbor the *HLA-DR3* gene, in contrast to 15–30% of the general population. We lack data on the associations between the HLA and HT, although HLA-DR3 is considered the major HLA antigen that predisposes to HT [56,65].

#### 4.1.2. Molecular Mechanisms That May Explain Predisposition to AITD concerning HLA

By binding to specific antigenic peptides, the HLA can recognize thyroid autoantigens as “foreign”, which causes an immune response that is mediated by autoreactive TLs (CD4+/CD8+). In the presence of environmental or infectious factors, this response is magnified and produced by TL and BL activation, which promotes the synthesis and secretion of cytokines and autoantibodies and causes AITD [67,68].

Similarly, the distal extracellular domain of HLA-II is an antigen-binding groove (containing AITD-associated amino acids); thus, any amino acid changes at these sites may alter the interaction between the antigen/HLA and the receptor of TLs, magnifying the immune response to foreign and self-antigens. Finally, it has been postulated that the elevated familial risk of GBD presupposes that there must be one or more associated genetic components in the case of HLA that must be linked to the degenerate motif in the *DRB1* gene product (that is, the amino acid at position 74 in the MHC II chain).

As a result, Arg and Glu are located at position 74 in the MHC molecule encoded by the GBD-associated DRB1*03 variant and the product of the protective variant DRB1*07; this position in the MHC chain is significant because it is within the P4 pocket, where the MHC peptide-binding motif overlaps with the TL receptor docking site [56,74].

HLA is one of the most prominent candidate genetic factors for several AIDs, because the major histocompatibility complex region is highly polymorphic and relevant to many immune response genes. Even so, the evaluation of different SNPs and alleles of the HLA gene has shown differences between the different populations studied (Asians, Caucasians, among others), and it is possible that the different HLA-genotyping methods remain impractical for analyzing large-scale associations (between different populations) [74].

#### 4.1.3. *PTPN22* Gene

After *HLA*, the *PTPN22* gene is the one that most predisposes to ADs. The *PTPN22* gene is located on chromosome 1p13.3–p13.1, and it encodes lymphoid-specific tyrosine phosphatase (LYP). LYP is capable of suppressing kinases that mediate TL activation and regulation, plays an important role in BL signaling, and is involved at multiple levels in the TL receptor signaling and activation cascade [75,76,77,78].

Thus, the minor allele 1858T in the *PTPN22* locus has a strong and consistent genetic association with AD. The cytosine changes to thymidine at nucleotide 1858, resulting in an amino acid change from arginine to tryptophan at codon 620 (R620W), which is located in the polyproline-binding motif P1. C1858T has been reported as a susceptibility locus associated with several ADs and AITD [75,76,77,78].

Some studies have found a significant association between the R620W (rs2476601) polymorphism (SNP) in *PTPN22* and an increased susceptibility to GBD and HT, which suggests that the *PTPT22* T allele could induce both diseases (Table 3).

The *PTPN22* R620W SNP was also found to increase the susceptibility to AITD in Caucasians and mixed races, but not in Asians. The susceptibility was independent of the thyroid autoantibody status [96].

#### 4.1.4. Molecular Mechanisms That Can Explain Susceptibility to AITD concerning *PTPN22* SNPs

The *PTPN22* gene can modulate the responses of TLs through the regulation of the antigen-presenting cell (APC) function, the downregulation of the Treg expansion at the peripheral level, or the transcriptional suppression of TLs (via transcription factors such as Foxp3). Additionally, *PTPN22* can influence the differentiation and proliferation of BLs, and it participates in the escape of autoreactive BLs to the periphery and in the enhancement of autoantibody development [97,98,99].

Moreover, LYP variants are characterized by the expression of losses or gains in their functions; thus, the C1858T variant optimizes the activity of PTPN22, and, as a consequence, an increase in TL and BL receptor signaling can occur, which modifies their functions and the secretion of cytokines [100].

Moreover, the *PTPN22* R620W SNP elicits a functional change in LYP such that the tryptophan-bearing LYP allele cannot bind the Csk, which causes the proliferation of TLs. Concomitantly, the levels of several Ig isotypes are increased. Among these antibodies, the levels of IgG and IgG4 are positively correlated with TPOAbs titers, and the levels of TPOAbs are positively correlated with the development of hypothyroidism and an increased inflammatory reaction [98,99,100,101].

Therefore, *PTPN22* has regulatory effects on several types of cells involved in the immune response and in different signaling pathways, increasing the risk of AITD.

#### 4.1.5. Cluster of Differentiation 40 (*CD40*) Gene

This gene is a member of the TNF-receptor superfamily. The encoded protein is a receptor on APCs, and it is essential for mediating a broad variety of immune and inflammatory responses. CD40 can be detected on APCs, and it is expressed in granulocytes, endothelial cells, smooth muscle cells, fibroblasts, and epithelial cells [102].

The interaction of CD40L with its respective receptor on BLs (CD40) is of critical importance for immunoglobulin isotype switching during the immune response. CD40L-induced signaling in these cells leads to the upregulation of adhesion and co-stimulatory molecules, as well as the production of proinflammatory cytokines, chemokines, growth factors, and matrix metalloproteinases [103,104].

Some *CD40* SNPs have suggested an increased risk of AITD, and especially for GBD (but not for HT). The best-studied SNP is rs1883832, which has been associated with a significant risk for GBD [105,106,107,108].

In addition, it was found that the frequencies of the CD40-1 C/C genotype and the C allele were significantly higher in individuals with GBD. For the SNP C64610G, the C/G genotype was significantly more frequent in HT, and the frequencies of the G allele in individuals with AITD were higher than in healthy subjects [109].

The association of the SNP *CD40* C/T-1 was evaluated in subjects with AITD, and a significant association with GBD was found, but not with HT. Individuals with the C⁄C or C/T genotype had a significantly higher risk of GBD than those with the T⁄T genotype, with no association with HT [110].

#### 4.1.6. Molecular Mechanisms That May Explain Susceptibility to AITD concerning *CD40* SNPs

CD40 signaling may potentially contribute to AITD in several ways: at the level of the TL selection phenomenon in the thymus, which allows autoreactive TL clones to escape deletion; in secondary lymphoid organs, where TLs are primed by BLs and other APCs; and in the thyroid, where CD40 signaling leads to the production of proinflammatory cytokines and chemokines that contribute to tissue destruction and inflammatory cell entry, with a potential increase in the risk of AITD [110,111].

#### 4.1.7. The Cytotoxic T Lymphocyte-Associated Factor 4 (*CTLA4*) Gene

CTLA4 is a negative regulator of the TL-mediated immune response, while CD152 is the product of the expression of the gene that codes for the synthesis of CTLA4. CTLA4 competes with the CD28 molecule (to bind to CD80 and CD86, which are co-stimulatory molecules on the surfaces of APCs). CD28 is constitutively expressed on TLs, and upon binding to CD80 and CD86, it emits a positive signal that results in TL activation [112].

When CTLA4 is expressed on the surfaces of TLs, it binds to CD80 and CD86 (with a higher affinity than CD28) and inhibits the positive signals of the CD28-CD80/CD86 interaction, causing independent negative signals and thereby limiting interleukin-2 production (IL-2) and TL proliferation and survival [112,113].

A CTLA4 malfunction in the endoplasmic reticulum leads to inefficient glycosylation and its reduced expression in TLs. This phenomenon can decrease the expression and function of CD152, which could be associated with the appearance of AITD [113,114,115].

Some studies have found a reduced expression of CD152 in individuals with HT, which suggests that TLs have defects in the expression of CD152 which abnormally activate TLs, leading to the increased secretion of thyroid autoantibodies [116,117,118,119,120].

In GBD, an increase in the expression of CD152 has been found in TLs, suggesting the defective function of CD152 (secondary to the SNPs of *CTLA4*). Research on the association between the SNPs of *CTLA4* and GBD found that different threonine/alanine transitions in the *CTLA4* gene lead to errors in its functioning in the endoplasmic reticulum, which cause an inefficient glycosylation reaction and a reduced expression of CTLA4 on the surfaces of TLs, decreasing their inhibitory function [119,121,122].

Other studies have investigated other *CTLA4* SNPs (rs231775 (A49G)), finding that carriers of the “G” allele have a higher risk of presenting AITD (relative to carriers of the “A” allele). Additionally, it was found that patients with type 1 diabetes mellitus who carried the “G” allele were also more likely to have AITD and that this variant was associated with a high risk for GBD [123,124,125,126,127,128].

An association between the SNP +49A/G and the risk of GBD has also been documented, and it was found that the “G” allele confers a significant risk for GBD compared to the “A” allele, which suggests that the *CTLA4* +49A/G SNP is associated with a genetic susceptibility to GBD [129,130].

Other studies have found that rs231779 has an association with AITD. Subjects with the “T” allele have a higher risk of hypothyroidism (compared with those with the “C” allele), although this variant has also been associated with GBD. Finally, other SNPs have been studied (rs5742909, rs231775); however, no significant associations have been found between these SNPs and AITD [131,132,133].

#### 4.1.8. Molecular Mechanisms That May Explain Susceptibility to AITD concerning *CTLA4* SNPs

CTLA4 has an inhibitory effect on immune responses that competes with the co-stimulatory molecules on APCs. The balance between the binding of CTLA4/CD28 to its common ligand (B7) plays an important role in determining the immune response. Therefore, factors that regulate the expression or activation of CTLA4 can affect this balance, with the consequent loss of control of the immune responses, which leads to autoimmunity. CTLA4 downregulates TL activation because CD152 expression on TLs increases in the late stage of immune activation (and competes with CD28 for binding to B7).

In the early stage of CD152 activation, CTLA4 binds to the intracellular domain and regulates the negative signaling that inhibits TL activation; consequently, the reduced CTLA4 expression can lead to a hyperactive and self-destructive immune response. Additionally, the variants described above can reduce the expression of the *CTLA4* gene and the amount of CTLA4 expressed in immune cells, although they can also change its structure, affecting its inhibitory effect on TL activation. This can translate into an uncontrolled immune response with the increased production and secretion of thyroid autoantibodies, and eventually, AITD [133,134].

#### 4.1.9. The *FOXP3* Gene

The *FOXP3* gene provides instructions for producing the forkhead box P3 (Foxp3) protein. The Foxp3 protein attaches (binds) to specific regions of DNA and helps control the activities of the genes that are involved in regulating the immune system. *FOXP3* is a key gene in the development of Tregs [135,136].

Several SNPs have been studied to evaluate their possible associations with AITD. For example, a meta-analysis revealed a possible association between AITD and the *FOXP3*-3279 SNP. For its part, the *FOXP3* (GT)n microsatellite has also been evaluated; however, no association with AITD was found [137].

Two meta-analyses evaluated the associations between the rs3761547, rs3761548, and rs3761549 SNPs and the susceptibility to GBD, indicating that the rs3761548 and rs3761549 SNPs were significantly associated with GBD susceptibility. In contrast, the rs3761547 SNP was not associated with GBD susceptibility. Moreover, rs3761548 was associated with GBD in Asians but not in Caucasians, whereas rs3761549 was associated with GBD in both Asians and Caucasians [138,139].

A study evaluated the associations of the SNPs of the *FOXP3* gene and HT; the rs3761548 SNP showed a significant association with HT. There were significantly higher serum levels of TPOAbs in the patients with the rs3761548 SNP [140].

The association of the functional SNPs of the *FOXP3* gene (−3499A/G, −3279C/A, and −2383C/T) with the prognosis of AITD was also evaluated, and it was found that the −3279CA genotype was more frequent in patients with GBD in remission than in patients with intractable GBD, and that the −2383CC genotype was more frequent in patients with severe HT than in those with mild HT [141].

#### 4.1.10. Molecular Mechanisms That Can Explain Susceptibility to AITD concerning *FOXP3* SNPs

Genetic variations in the *FOXP3* gene may promote AITD by weakening the inhibitory function of Tregs and promoting an autoimmune response. Upon its expression, a self-regulating transcriptional circuit stabilizes the expression of *FOXP3* to consolidate the differentiation of Tregs and activate the suppressive function. Therefore, in AITD, autoreactive TLs are more resistant to suppression, not so much because of the low number of Tregs but because of their regulatory inability. Likewise, in the loss of immune tolerance, alterations in *FOXP3* acetylation could disrupt the transcriptional and epigenetic regulation of *FOXP3*, which results in less Treg generation and a weaker suppressor function. In this sense, a deficiency of Tregs (rather than an absolute numerical deficiency) could be considered, where a functional deficit would better explain its association with AITD [142,143].

#### 4.1.11. α Chain of IL-2R (*IL-2Rα*) Gene

The *IL-2Rα* gene is located on chromosome 10, and it was the first to be defined at the molecular level due to its unique ability to independently bind IL-2. IL-2Rα (also known as CD25) is a glycoprotein that plays an essential role in the TL response to IL-2, which is the main growth factor for these cells (CD25 expression is important for proliferation, longer life expectancy, and TL function) [144].

CD25 occurs on the surfaces of maturing TLs and BLs, undergoes transient expression on activated TLs and BLs, and occurs constitutively on Tregs, which inhibit the activation of autoreactive TLs [145].

It has been shown that the SNPs of this gene occur in people with AITD; thus, some studies found that the *IL-2Rα* SNP (rs7090369, allele T) was more frequent in patients with AITD (the TT genotype induces a statistically significant (5.2 times) higher risk) [146].

These results contrast with other discordant findings, where no associations between *IL-2Rα* SNPs and the presence of AITD were found [147,148,149,150,151].

#### 4.1.12. Molecular Mechanisms That May Explain the Susceptibility to AITD concerning *IL-2Rα* SNPs

Given the “quantal theory” of TL activation, the cells of the immune system recognize and react to different antigens (self and foreign), proliferating and differentiating into effector cells in an all-or-nothing (quantum) manner. In addition, the theory establishes that these cells make this decision only after “counting” the number of receptors for the antigen that has been activated, which ultimately determines the number of activated IL-2R molecules, and this number is what determines the quantum decision to progress through the cell cycle and undergo DNA replication and the related cytokinesis, which is the basis for clonal expansion. According to the above, there are several IL-2R molecules expressed on the TL surface that are considered “critical” for the cellular response to stimuli; thus, any potential epigenetic modifications in the promoter region of this gene could affect the expression of the gene and therefore constitute a possible regulatory mechanism. For instance, the hypomethylation within the *IL-2Rα* promoter could alter the IL-2Rα expression on Tregs, which competitively binds IL-2 and thereby plays a key role in the development and immunosuppressive function of Tregs. Thus, epigenetic alterations in the region of the *IL-2Rα* may explain its association with AITD; however, these alterations do not necessarily extrapolate to both extremes of AITD, as they appear to be more prevalent in individuals with GBD than in patients with HT, which is probably due to the underlying mechanisms in one disease or the other [152,153].

### 4.2. Thyroid-Specific AITD Susceptibility Genes

Although AITD has been associated with immunomodulatory genes, this does not fully explain the specific autoimmune component directed toward the thyroid. Therefore, antigen-specific genes (*TSHR*, *Tg*, *TPO*, among others) are a group of candidate genes that are potentially associated with AITD.

#### 4.2.1. The *TSHR* Gene

TSHR is encoded by a gene located at 14q31 and is part of the glycoprotein hormone receptors, which are a subgroup of G protein-coupled receptors (class A) [154,155,156,157].

TSHR has a large extracellular domain, seven transmembrane domains, and a small intracellular domain. The endogenous TSHR ligand is TSH; therefore, such binding activates several coupled signaling pathways, which promote the expression of “effector” genes that control the growth and differentiation of thyrocytes and the synthesis and secretion of thyroid hormones [156,157,158].

TSHR is considered one of the major thyroid autoantigens, specifically in GBD. In the presence of its autoantibody (TRAb), the activation of signaling cascades is stimulated, simulating the effect of constant TSH stimulation on the thyroid, which clinically results in hyperthyroidism, although biologically, there are other types of stimuli for TSHR activation (for example, through the autonomous activation of the TSHR (induced by somatic or germline mutations in the TSHR gene) and thyrostimulin) [159,160].

Although thyrostimulin is a potent stimulator of the thyrocyte function, its influence on thyroid physiology is not fully understood, although it likely plays a paracrine role in the anterior pituitary, and in other tissues that express the TSHR [160,161,162].

Some studies have evaluated possible associations between different *TSHR* SNPs and the AITD risk. For instance, one study combined a panel of 98 SNPs in patients with GBD. In total, 28 SNPs revealed significant associations with GBD, with the strongest SNP associations at rs179247 and rs12101255, which are both located in intron 1 of the *TSHR* gene [163].

Another study determined five *TSHR* SNPs (rs179247, rs12101255, rs2268475, rs1990595, and rs3783938) in patients with AITD. The frequencies of the T allele and TT genotype of rs12101255 in GBD patients were significantly higher. The frequency of the A allele of rs3783938 was also significantly higher in HT patients. The AT haplotype (rs179247-rs12101255) was significantly associated with an increased risk of GBD, and the A allele of rs179247 was associated with ophthalmopathy [164].

Possible associations have also been evaluated between GBD and rs179247 or rs12101255 (in GBD), with both SNPs associated with a significant increase in GBD risk. No association was found between SNP rs179247 and ophthalmopathy [165].

In the same direction, *TSHR* SNPs (rs179247, rs12101255, and rs2268458) were evaluated in relation to the risk of GBD. rs179247 and rs12101255 were significantly associated with GBD [166].

Another study evaluated participants with GBD and found that the AA genotype for rs179247 significantly increased the risk for GBD, whereas the GG genotype for rs12885526 increased the risk for ophthalmopathy [167].

Moreover, the associations of two *TSHR* SNPs (rs179247 and rs12101255) in three independent European cohorts (in participants with GBD) were validated. Both SNPs showed strong associations with GBD (in all three cohorts) [168].

These same SNPs (rs179247 and rs12101255) were evaluated in patients with GBD, and they both had significant associations with GBD in the Asian, European, and South American subgroups [169].

#### 4.2.2. Molecular Mechanisms That May Explain Predisposition to AITD concerning *TSHR* SNPs

Several mechanisms may explain the associations between *THSR* SNPs and AITD, specifically in GBD. For instance, two mechanisms modify the peripheral tolerance and central tolerance. One of the mechanisms proposes that intron 1 SNPs associated with GBD participate in the regulation of the alternative splicing of the TSHR mRNA in the thyroid. Because the carriers of the risk alleles for GBD have higher relative expressions of some variants, such as ST4 and ST5 (soluble isoforms), these isoforms may have greater immunogenic potentials, as they are associated with the loss of peripheral tolerance [170].

The other mechanism is due to the modulation of the expression of the TSHR in the thymus, which highlights the role of central tolerance, and it is influenced by the expressions of autoantigens (in this case, the TSHR) inside the thymus (in the process of the negative selection of clones of autoreactive TLs); therefore, the *TSHR* SNPs could influence the level of the expression itself in the thymus, which is a potential trigger of thyroid autoimmunity. Additionally, various genetic–epigenetic interactions involving different *TSHR* SNPs also regulate the *TSHR* gene expression in the thymus, allowing the escape of autoreactive TLs that can recognize the TSHR and induce GBD. This mechanism is based on the presence of the protective allele of the SNP rs179247, and it has been associated with increased TSHR mRNA expression in the thymus (but not in the thyroid). Therefore, this mechanism could potentially favor the negative selection of self-reactive TLs for the TSHR. Thus, any failure in the presentation of the TSHR by the APCs (in the thymus) will induce a failure in the central tolerance towards the TSHR [171,172,173].

#### 4.2.3. The *Tg* Gene

The *Tg* gene is located on chromosome 8q24. This gene contains more than 16,000 SNPs, and around 10 of them are considered pathogenic, as they are found in germ cells. This characteristic suggests that this gene could be associated with AITD [174].

Tg is a hyperglycosylated protein that is expressed in thyrocytes and secreted into the follicular lumen, where it accumulates. Dimeric Tg undergoes an iodination process to form different tyrosine residues. This process is regulated by iodine intake from the diet. Once iodinated, Tg is transported to the cytosol to be subsequently metabolized, releasing T3 and T4. Therefore, Tg is a precursor for the synthesis of thyroid hormones. TSH and the transporter protein responsible for storing iodine in the colloid intervene in this process [175,176,177].

Some *Tg* SNPs and allelic variations have been associated with AITD. For example, a study carried out on 56 families revealed seven major loci and other minor loci for AITD. One of the minor loci was on chromosome 8q24. Additionally, a microsatellite was found within intron 27 of the *Tg* gene. This microsatellite showed a strong association with AITD [178].

Another study carried out on 102 families with AITD found seven loci associated with AITD: three of them (on chromosomes 6p, 8q, and 10q) showed associations with AITD, another three showed associations with GBD (7q, 14q, and 20q), and one locus showed an association with HT (12q) [49].

In one study in patients with AITD, the researchers used microsatellite markers located in the 8q24 region, Tgms1 and Tgms2, microsatellite markers in introns 10 and 27, and an SNP in exon 33 of *Tg*. No differences in the allele frequencies were observed; however, in the patients with HT, a significant association between the 330 bp/352 bp genotype of Tgms2 and HT was found [179].

Three different *Tg* SNPs were evaluated in subjects with AITD (E10, E12, E33). The genotype and allele frequencies at E10SNP158, E12SNP, and E33SNP in the GBD patients showed significant differences in the T/T genotype of E33SNP and the G/G genotype of E12SNP. It was also found that the E33SNP T/T genotype was positively associated with the development of GBD, whereas the E12SNP G/G genotype protected against it [180].

The occurrence of four common *Tg* SNPs (E10SNP24 T/G and E10SNP158 T/C in exon 10, E12SNP A/G in exon 12, and E33SNP C/T in exon 33) was evaluated in subjects with AITD. There were no differences in the allele or genotype frequencies. The haplotype analysis revealed that the G-C-A-C haplotype was significantly associated with HT and TgAbs positives [181].

A panel of 25 SNPs across an extended 260 kb region of the *Tg* gene in subjects with AITD was also explored. Five SNPs revealed significant associations with GBD, with the strongest SNP associations at rs2256366 and rs2687836, which are both located in intron 41 [182].

Another study on subjects with AITD evaluated SNPs in exons 10, 12, and 33 of the *Tg* gene (four Tg SNPs: E10SNP24 T/G, E10SNP158 T/C, E12SNP A/G, and E33SNP C/T). Several allele and genotype frequencies differed within the AITD group. Additionally, a statistically significant difference in the frequencies of the selected *Tg* SNP haplotypes was present among the AITD patients [183].

In addition, the following *Tg* SNPs were genotyped in patients with AITD: rs2069566; rs2076739; rs121912646; rs121912647; rs121912648; rs121912649; rs121912650; rs137854433; rs137854434; and rs180195. It was found that the variant 1623 A/G SNP (rs180195) is a marker for thyroid autoimmunity. Moreover, the authors found a significant difference in the distribution of the major allele (G) vs. the minor allele (A) in the patients with HT. The genotype homozygous AA and heterozygous GA rs180195 SNPs were more closely associated with AITD [184].

Similarly, the following SNPs were genotyped in patients with AITD: rs180195G/A; rs853326G/A; rs2076740C/T; rs2703013G/T; rs2958692C/T; rs733735A/G. The rs180195 GG genotype was more frequent in the HT patients, and the rs2703013 TT genotype was less frequent in the AITD patients. In the rs2958692 SNP, the T allele was significantly more frequent in intractable GBD than in GBD in remission [185].

Finally, the associations between the rs2076740, rs853326, rs180223, and rs2069550 *Tg* SNPs and AITD have also been quantified. There were no significant associations found between the rs2069550, rs180223, and rs853326 SNPs and the AITD risk [186].

#### 4.2.4. Molecular Mechanisms That May Explain Predisposition to AITD concerning *Tg* SNPs

*Tg* variants may predispose to AITD by altering Tg degradation in endosomes, which may give rise to a repertoire of pathogenic Tg peptides. Genetic interactions between HLA-DRβ-Arg74 and some *Tg* variants have been shown to increase the risk of presenting GBD. Additionally, it has also been shown that some Tg peptides can bind to the HLA-DRβ-Arg74 pockets, with one of these peptides being a major epitope of TLs. It should also be noted that Tg is an important target in the iodine-induced autoimmune response (and iodine is an essential component of Tg). Thus, in experimental autoimmune thyroiditis, Tg iodination can induce an increase in the TgAbs levels, which is probably due to the structural modification of Tg, inducing a greater antigenicity of the molecule (facilitating the selective presentation of cryptic peptides of Tg to APCs) [187,188].

#### 4.2.5. The *TPO* Gene

The *TPO* gene is located on chromosome 2p25 and encodes a glycosylated hemoprotein of 933 amino acids. The full-length TPO (TPO 1) protein, which consists of 933 amino acids, contains a large extracellular domain, a short transmembrane domain, and an intracellular C-terminal region [189,190].

Even though TPO is considered one of the major thyroid antigens, there are not many studies that evaluate *TPO* SNPs and their possible associations with AITD. For instance, the association between the T1936C, T2229C, and A2257C TPO SNPs and TPOAb levels was evaluated, and it was found that, in the presence of the C allele of T1936C, the TPOAb levels were significantly increased [191].

Another study evaluated the association between genetic defects in the *TPO* gene and patients with hypothyroidism (and TPOAb negatives). Six different SNPs were identified, as well as two novel deletions. Two of the six SNPs revealed significant associations with hypothyroidism (Thr725Pro (rs732609) and Asp666Asp (rs1126797)). The c.2173C allele of Thr725Pro showed a significant association among hypothyroid patients [192].

Some genetic variants associated with TPOAbs are also involved in HT development. For instance, three genetic variants have shown associations with HT: rs10774625 (the *ATXN2* gene), rs7171171 (near the *RASGRP1* gene), and rs11675434 (the *TPO* gene). Among them, rs1077462 and rs11675434 have shown significant associations with TPOAb levels, and rs7171171 has been associated with the thyroid size [193].

Eight SNPs in the *TPO* gene were genotyped to evaluate the association with the development, severity, and intractability of AITD. The rs2071400 T carriers (CT + TT genotypes) and rs2071403 GG genotypes were more frequent in AITD. The serum levels of TPOAbs were significantly higher in AITD patients with rs2071400 T carriers (CT + TT genotypes) than in those with the CC genotype, and they were also significantly higher in AITD patients with rs2048722 T carriers (CT + TT genotypes) than in those with the CC genotype [194].

The relationships between some *TPO* SNPs and the TPOAb levels in patients with subclinical hypothyroidism (SCH) were evaluated. The TPOAb levels (and genotypes A2095C and A2173C) were significantly higher and more frequent in the subjects with SCH. The risk of developing SCH in the individuals with C alleles was higher than in the individuals without these alleles in the A2095C and A2173C regions, respectively [195].

Another study evaluated the association of several *TPO* SNPs and the TPOAb levels in patients with autoimmune hypothyroidism. The TT genotype of rs2071400 C/T and the T allele (and the rs732609 A/C SNP (CC genotype and C allele)) were significantly more frequent in patients with SCH and overt hypothyroidism, and there was a significant difference in the CC genotypes and C alleles between the SCH and overt hypothyroidism patients [196].

#### 4.2.6. Molecular Mechanisms That May Explain Predisposition to AITD concerning *TPO* SNPs

Genetic studies suggest that *TPO* gene mutations with autosomal recessive inheritance are one of the most common causes of AITD, with several different inactivating mutations identified in patients with total iodide organification defects. AITD may result from several mechanisms, including the total absence of TPO activity, the inability of TPO to bind to the heme cofactor, the inability to interact with the Tg substrate, and abnormal subcellular localization. Although TPOAbs are valid clinical biomarkers of AITD, they are generally considered to be secondary to the thyroid damage inflicted by TLs [197].

#### 4.2.7. Other Susceptibility Genes for AITD

Other candidate genes involved in the risk for AITD have been described; however, the results have been conflicting and, in some cases, associations have only been found with GBD or HT (Table 4).

## 5. Epigenetic Mechanisms in AITD

**Figure 3:**
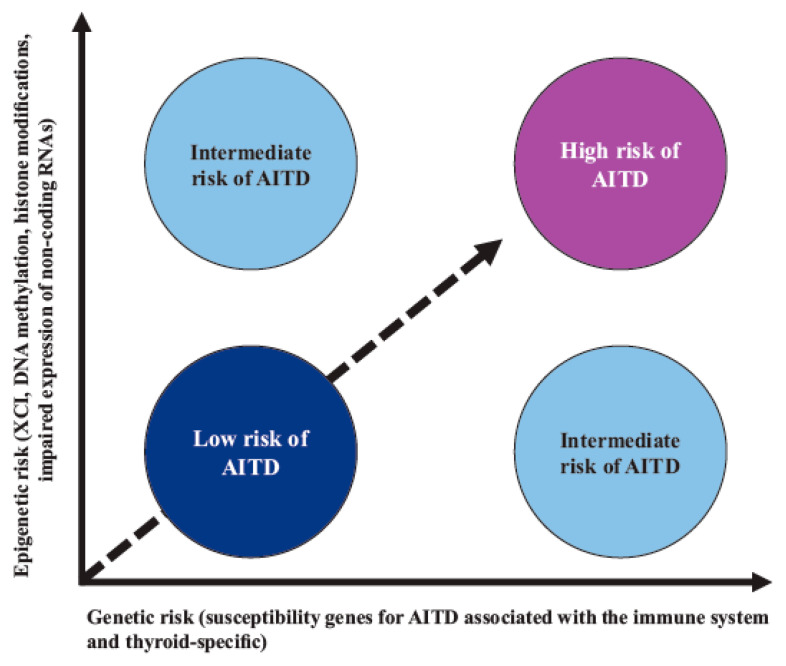
Interaction between genetic susceptibility and epigenetic factors in AITD. Genetic risk by itself confers a low-moderate risk for AITD; however, risk is increased when there is synergy with epigenetic modifications of regulatory regions that are capable of controlling gene expression. Abbreviations: AITD: autoimmune thyroid disease, XCI: X-chromosome inactivation.

In AITD, studies have shown that the vast majority of the “candidate” gene SNPs are associated with a given risk for the disease; however, this risk is low, indicating that, for a given genetic risk factor, other nongenetic (epigenetic) modifiers are probably necessary to trigger AITD (Figure 3) [212,213,214].

Among the epigenetic factors associated with AITD, the skewed X-chromosome inactivation (XCI) SNPs of the genes involved in DNA methylation, the DNA methyltransferases (DNMTs) genes, or the methylenetetrahydrofolate reductase (MTHFR) and methionine synthase reductase (MTRR) genes stand out. Other SNPs have also been implicated, such as the immunoregulatory factors ADRB2 (hypermethylated), ICAM1 (hypomethylated), and B3GNT2 (hypermethylated), as well as histone modifications and the impaired expression of noncoding RNAs, among other factors [215,216].

### Molecular Mechanisms That May Explain Predisposition to AITD concerning Epigenetic Factors

XCI occurs early in development and could lead to the mature TL population that is not sufficiently educated to recognize the components of XCI as self during thymic education. Subsequently, when the components of the XCI are encountered later in life, they could be recognized as nonself, which leads to an autoimmune response that cross-reacts, which causes AITD [217,218].

DNA methylation studies are limited, and there is high variability among the individuals studied; nonetheless, it could be argued that abnormal DNA methylation is involved in the pathogenesis of AITD. Some genetic SNPs of the DNA methylation-regulating genes can also cause dysfunction of these genes and aberrant DNA methylation, which further increases host susceptibility to AITD [219].

Moreover, histone modifications play important roles in controlling chromatin compaction, nucleosome dynamics, and DNA repair, and they can directly regulate transcription. Similar to DNA methylation, histone modifications are highly dynamic, and it has been suggested that they play a role in the modulation of immune tolerance and AITD [220,221].

Further, it has been found that the gene promoter methylation in patients with AITD is coupled to changes in the chromatin structure that allow the silencing of the gene expression [222,223].

Moreover, some associations have been found between the impaired expressions of noncoding RNAs and AITD. For instance, micro-RNAs (miRNAs) are small RNA molecules that target about 60% of all genes and interact with other epigenetic mechanisms, such as DNA methylation and histone tail modifications. Some miRNAs play important roles in regulating the immune function and maintaining immune homeostasis, such as miR-223-3p and miR-155-5p; therefore, the abnormal expressions of miRNAs involving the immune function can potentially contribute to the development of AITD [224].

miRNAs play a crucial regulatory role in Toll-like receptor signaling, which results in the activation of the NF-κB, IRF, and AP-1 transcription factors, which regulate the expression of proinflammatory cytokines. This indicates that miRNAs participate in the regulation of the gene and autoimmune antibody expression, contributing to the occurrence of AITD [224,225].

Despite the biological plausibility of the association between genetics and epigenetics in AITD, the study results have focused primarily on GBD patients and are limited to single-gene studies. Moreover, the available studies have largely descriptive designs, which makes it difficult to demonstrate the association between these factors and the presence of AITD (Table 5).

## 6. Nongenetic Factors in AITD

### 6.1. Role of Nongenetic (and Infectious) Factors in AITD

A significant number of nongenetic factors have been associated with AITD, which can be divided into infectious and noninfectious. The infectious factors have almost always been evaluated retrospectively (based on the measurement of antibodies against microorganisms), especially for *Y. enterocolitica*, *H. pylori*, *B. burgdorferi*, Hepatitis C virus (HCV), Hantavirus, Saccharomyces, *T. gondii*, human immunodeficiency virus (HIV), and the gut microbiota [99].

#### Molecular Mechanisms That May Explain Predisposition to AITD concerning Nongenetic (Infectious) Factors

The pathophysiological mechanisms that explain possible associations between infectious factors and AITD are still unclear [230,231,232,233]. These mechanisms are summarized in Table 6 and include the following:

- Molecular mimicry (the activation of autoreactive TLs by microorganism peptides that are structurally similar to self-peptides);
- Viral and bacterial superantigens (the activation of autoreactive TLs that express particular Vβ segments) and the enhanced processing and presentation of autoantigens (by APCs recruited to an inflammatory site and followed by autoreactive lymphocyte priming);
- The bystander effect (enhanced cytokine production that induces the expansion of autoreactive TLs);
- The activation of lymphocytes by lymphotropic viruses (an infection of BLs resulting in BL proliferation and excess antibody production);
- The formation of circulating immune complexes and cytokine storms.

### 6.2. Role of Nongenetic (and Noninfectious) Factors in AITD

These factors can be classified as nutritional or nonnutritional. The nutritional factors associated with AITD include iron deficiency, iodine excess, selenium deficiency, vitamin D deficiency, and gluten consumption [255]. The effects of the excess or deficiency of these nutritional factors and their possible molecular mechanisms related to AITD are summarized in Table 7.

Among the nongenetic and nonnutritional factors, endocrine disruptors, including smoking, alcohol consumption, psychological stress, and drugs such as amiodarone, IFN-α, and fetal microchimerism, among others, stand out. The molecular mechanisms that explain the associations between these factors and AITD are complex and not entirely clear. These factors most likely interact with an individual’s genetic susceptibility to manifest a given AITD risk [267,268,269]. The molecular mechanisms are summarized in Table 8.

### 6.3. Other Nongenetic and Nonnutritional Factors Related to AITD

Fetal cell microchimerism (FCM) and maternal cell microchimerism (MCM) are phenomena that occur during pregnancy, in which the export of cells, including some from the immune systems, can occur either from the fetus to the mother (FCM) or vice versa (MCM) [294].

This implies that an individual can have a specific proportion of cells from a genetically different organism. This bidirectional traffic of transplacental cells begins in the second week of gestation and increases as the pregnancy progresses. These allogeneic cells can persist in the host for years or decades [295,296].

One way to determine the presence of FCM is by demonstrating the presence of male cells in a woman with a previous male pregnancy (employing polymerase chain reaction or fluorescence in situ hybridization analysis) [297,298].

Studies related to AITD have been conflicting, as some have found increased numbers of circulating fetal cells (tissue and peripheral levels), whereas, in other studies, it has been reported that the number of fetal cells is higher in individuals without AITD (suggesting a protective role for AITD) [297,298,299,300].

#### Molecular Mechanisms That May Explain Predisposition to AITD concerning FCM and MCM

The association between FCM and AITD can be explained in various ways. Some hypotheses have been raised, and one of them suggests that FCM induces a graft-versus-host reaction, which may be mediated by infectious or environmental factors, drugs, or abnormal tissue proteins, among others, and which breaks the maternal tolerance to fetal cells and results in AITD [301].

For this response to occur, three conditions must be met. The first is that fetal cells must be present at the site of the immune reaction (blood and thyroid). The second is that the microchimeric cells must be TLs or BLs (immunologically competent). The third is that the microchimeric cells are capable of recognizing host cells as foreign [301,302].

Another hypothesis is that the fetal microchimeric cells are recognized by the mother as “foreign”, and because the fetal cells contain paternal genes, the cells are “semi-foreign” to the mother so that, after delivery, the maternal cells can react against paternal antigens on microchimeric cells within the thyroid, either through a direct response to the microchimeric cells or by cross-reactivity (“molecular mimicry”). Additionally, it may also be the case that fetal APCs may present maternal antigens to maternal immune cells, which causes an immune response from the mother against her cells [303,304].

Moreover, a possible “protective” effect of FCM on the presence of AITD has also been described, which can be explained because microchimeric cells are potential “repairers” of injured or damaged tissues. For this, fetal cells must migrate to the damaged or injured area and should show plasticity [305,306].

Finally, a hypothesis that states that FCMs are innocent bystanders and do not participate in the triggering or exacerbation of AITD (which suggests that the fetal cells are merely a “remnant” of the pregnancy) [307,308,309,310].

## 7. Thyroid Autoantibodies

The primary feature in the pathogenesis of AITD is the activation of both humoral and cellular immune responses against an autoantigen. Thus, autoantibodies directed against various thyroid antigens contribute to the expansion of the autoimmune response, and they even play a functional role as they can induce the secretion of thyroid hormones (such as TRAbs). The major thyroid antigens that can induce an antibody-mediated response are the TSHR, Tg, and TPO; however, two other antigens are also described: pendrin and the Na/I symporter (NIS) [311].

The main features of these “major” thyroid antigens are summarized in Table 9.

### 7.1. TRAbs

TRAbs are IgG-type antibodies, and they can be classified into two classes: those that stimulate the TSH receptor (TSAbs) and those that block it (TBAbs). TSAbs stimulate the TSHR and are highly prevalent in GBD patients. TBAbs increase the risk of hypothyroidism and are detectable in between 10 and 90% of individuals with HT, although they can also be found in a minority of GBD patients. TRAbs have also been detected that can bind to the TSHR but do not alter thyroid function; thus, these TRAbs are neutral (TNAbs) [312,313,314].

### 7.2. TPOAbs

There is no convincing evidence that indicates that TPOAbs initiate the phenomenon of thyroid autoimmunity. This has a simple explanation, which is that these antibodies cannot penetrate the tight junctions between thyroid cells, and therefore they cannot bind to their autoantigen (TPO), which is located apically. However, they could play a role in the autoimmune response in cases in which the tight junctions are disrupted as a consequence of TL-mediated damage [315].

However, TPOAbs may predispose to tissue destruction (mediated by the complement pathway and by antibody-dependent cell-mediated cytotoxicity). Thyroid cells under the complement effect are capable of releasing proinflammatory molecules, increasing tissue destruction and the inflammatory reaction, and inducing a greater risk of AITD [316,317].

### 7.3. TgAbs

The role of TgAbs in the pathogenesis of AITD is less conclusive than those found with TRAbs and TPOAbs; however, more than 90% of HT patients are TgAbs-positive. One explanatory hypothesis is antibody-dependent cell-mediated cytotoxicity, in which cells containing the antigen are killed by TLs (NK) and macrophages. Furthermore, in animal models of AITD, TgAbs are detected earlier (leading to the subsequent discovery of the presence of TPOAbs), which suggests that the tolerances of BLs and TLs are likely initially altered towards Tg and later towards TPO [41,318].

Likewise, universal salt iodization programs have discovered that, in individuals with excessive iodine intake, the frequency of thyroid autoimmunity is greater. This excess of iodine is capable of modifying the three-dimensional structure of Tg (probably mediated by the phenomenon of Tg proteolysis, which is induced by reactive oxygen species). As a consequence, the autoantigenicity of Tg is increased, which results in a greater response of autoreactive TLs and a greater generation of TgAbs [319].

### 7.4. Pendrin and NIS Antibodies

The role of pendrin and NIS antibodies in the pathogenesis of AITD is conflicting, which suggests that these antibodies are present in some patients with AITD, notwithstanding that their clinical importance in the pathogenesis of AITD and on thyroid function is yet to be determined. So far, their measurement does not seem to offer any diagnostic, treatment, or prognostic benefits [320,321].

Finally, a direct toxic effect of the thyroid Abs on extrathyroid organs has also been proposed, for example, in individuals with encephalopathy due to Hashimoto’s thyroiditis; however, although it is also clear that both TPOAbs and TgAbs have been found in cerebrospinal fluid in subjects with HT, a strong association between the presence of Abs and the clinical manifestations of the disease has not been demonstrated [322].

## 8. Summary of Molecular Mechanisms Leading to AITD

AITD is triggered by a variety of factors (genetic, nongenetic, epigenetic, and environmental). Among the susceptibility genes associated with the immune system, the following stand out: *HLA-DR3*; *PTPN22*; *CD40*; *FOXP3*; *CTLA-4*; and *IL-2Rα*, although thyroid-specific susceptibility genes have been described (*TSHR*, *Tg*, and *TPO*). Some SNPs in these genes play key roles that help explain (at least in part) the increased risk for AITD. For example, the SNPs in *FOXP3* and *IL-2Rα* are involved in peripheral tolerance mechanisms, while the SNPs in the *CD40*, *CTLA-4*, and *HLA* genes compromise the activation of TLs and the antigenic presentation; therefore, the SNPs in immunoregulatory genes can potentially alter the functioning or normal development of the central and peripheral tolerance mechanisms and the interaction of TLs with APCs. Some SNPs in other genes involved in the synthesis of cytokines with potential inflammatory effects have also been described and associated with an increased risk of AITD.

Although genetic susceptibility can potentially explain the pathogenesis of AITD, the genetic risk by itself is low; however, this risk is increased when there is synergism with some components or epigenetic modifications in the region’s regulators that are capable of controlling the gene expression. These epigenetic modifications include the XCI SNPs of the genes involved in DNA methylation, DNMT genes, or MTHFR and MTRR genes. Histone modifications and the impaired expressions of noncoding RNAs have also been implicated.

Environmental factors can be infectious and noninfectious and, in turn, nutritional and nonnutritional. These factors can, by mechanisms not yet elucidated, increase the susceptibility to AITD. However, the key point for the development of AITD is the infiltration of the thyroid by APCs, which may be induced by environmental factors.

Considering that the thyroid follicular cells from individuals with AITD can also abnormally express HLA-II (induced by IFN-γ), the phenomenon of thyroid autoantigen presentation, which facilitates the activation of TLs, is feasible. Likewise, there is also the thyroid infiltration of BLs, cytotoxic TLs, and TLs (CD4+). The interaction with APCs leads to the activation of TLs (CD4+) and the differentiation towards Tregs and Th (Th1, Th2, and Th17), with an imbalance in the Th1:Th2 ratio. For HT, the predominance is towards a Th1 response, whereas in GBD, the predominance is towards a Th2 response.

**Figure 4:**
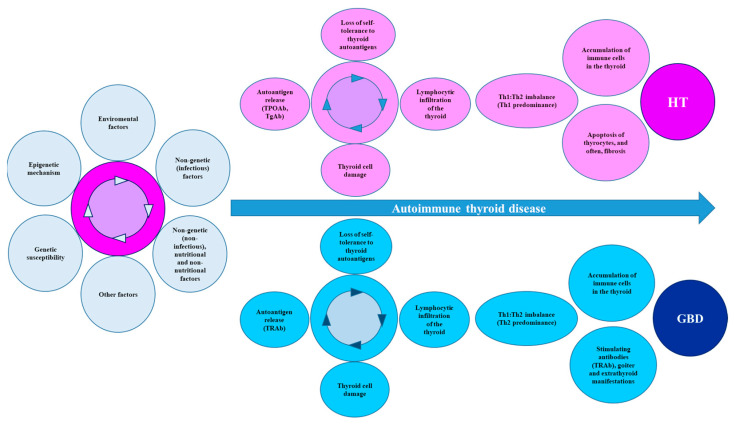
Summary of the mechanisms that lead to AITD. AITD is the product of multiple environmental factors that act on the basis of genetic susceptibility, together with some epigenetic mechanisms, leading to a loss of immune tolerance, with destruction of thyroid tissue and increased synthesis and secretion of autoantibodies. Finally, the Th1:Th2 imbalance directs the clinical and biochemical manifestations towards HT or GBD. Abbreviations: AITD: autoimmune thyroid disease, GBD: Graves-Basedow disease, HT: Hashimoto’s disease, Tg: thyroglobulin, TgAb: Tg autoantibodies, TPO: thyroid peroxidase, TPOAbs: TPO autoantibodies, TRAbs: thyroid stimulating hormone receptor autoantibodies.

In addition, an attenuated Treg response has also been found, which may increase the proinflammatory activity of Th17. These mechanisms involve cytokines/chemokines and/or cytotoxins. For HT, apoptosis and subsequent fibrosis lead to the presence of hypothyroidism, while in GBD, the persistent stimulation of the TSHR by its autoantibody (TRAb) induces hyperthyroidism, goiter, and extrathyroidal manifestations (Figure 4).

## Figures and Tables

**Table 1:** Animal models of AITD.

Results and Associated Mechanisms	References
The frequency of AITD is about 100% when the NOD mice are exposed to 0.05% NaI in drinking water (between six and eight weeks after its treatment), and symptoms of chronic inflammation are discovered three to four weeks later (an effect likely due to iodide-induced oxidative stress)	[22,23]
All animals that have thyroid dysfunction or lesions are capable of producing the Tg-specific antibodies IgG1 and IgG2b	[23,24]
Both Th1 and Th2 cytokines are expressed in the thyroid of these mice after NaI administration, reaching their nadir between the fourth and sixth week (for Th1) and between the eighth and fifteenth week (for Th2) after administration, that is, when the tissue damage is at its worst and is in the chronic phase	[24,25,26]
The thyroid cellular infiltrate is represented by TL CD4+ and TL CD8+, BL, macrophages, natural killer cells, and DCs. Cellular infiltration begins with TL CD4+, followed by TL CD8+, macrophages, and later, by BL	[27,28]
The complete elimination of the TL CD4+ and TL CD8+ prevents the appearance of AITD by suppressing the tissue infiltration of TL and the production of autoantibodies; in addition, the secretion of cytokines that have a high inflammatory power (IFN-γ and TNF-α) and the lysis of thyrocytes mediated by perforin/granzyme are reduced	[29,30,31]
Following Tg stimulation, invariant natural killer cells produce cytokines (IFN-γ, TNF-α, IL-2, IL-4, and IL-10), suggesting a role (at least indirectly) in the susceptibility and maintenance of the immune response in AITD	[31,32]
Depletion of Treg (CD25+) increases the severity of thyroiditis	[32,33,34]
Some TL helper (Th), including Th1, Th2 and Th17, express transcription factors (T-BET, GATA-3 and RORγt) and secrete cytokines (IFN-γ, IL-4, IL-13 and IL-17), all they are involved in the inflammatory phenomenon and in the synthesis of autoantibodies	[31,35,36]
Depletion of BL through anti-CD20 monoclonal antibodies decreases the severity of the disease by reducing the levels of thyroid autoantibodies. Therefore, if one considers that BL can secrete inflammatory cytokines and that they can act as antigens, presenting cells and promoting the activation and expansion of autoreactive TL, then it is understandable that BL has a role in the pathogenesis of the AITD	[37,38,39]
The increase in the immunogenicity of TPO and Tg is associated with a loss of tolerance towards both molecules, which promotes the generation of many peptides, inducing greater processing by MHC molecules and greater presentation to autoreactive TL	[40,41,42]

Abbreviations: AITD: autoimmune thyroid disease, BL: B lymphocyte, DCs: dendritic cells, IFN: interferon, Ig: immunoglobulin, IL: interleukin, NOD: non-obese diabetic, Tg: thyroglobulin, Th: T helper lymphocyte, TL: T lymphocyte, TNF: tumor necrosis factor, Treg: regulatory T lymphocyte.

**Table 2:** Recent studies on SNPs and HLA alleles in individuals with AITD.

HLA Typing Procedures	Method	Populations	AITD Type (Ref)	Results
HLA genotyping (*HLA-A*, *HLA-B*, *HLA-C*, *HLA-DQB1*, and *HLA-DRB1*)	Next-generation sequencing (NGS)	Caucasians	GBD [72]	The following alleles were associated with increased risk of GBD: HLA-B*08:01, HLA-B*39:06, HLA-B*37:01, HLA-C*07:01, HLA-C*14:02, HLA-C*03:02, HLA-C*17:01, HLA-DRB1*03:01, HLA-DRB1*11:01, HLA-DRB1*13:03, HLA-DRB1*01:03, HLA-DRB1*14:01, HLA-DQB1*03:01, and HLA-DQB1*02:01. The alleles HLA-B*39:06, HLA-B*37:01, HLA-C*14:02, HLA-C*03:02, HLA-C*17:01, and HLA-DRB1*14:01 were novel GBD-associated. Moreover, the frequencies of HLA-B*07:02, HLA-C*07:02, HLA-C*03:04, HLA-DRB1*07:01, HLA-DQB1*02:02, and HLA-DQB1*03:03 were significantly lower in GBD (compared to controls).
HLA genotyping (*HLA-A*, *HLA-B*, *HLA-C*, *HLA-DRB1*, and *HLA-DQB1*)	DNA extraction and sequence-based typing for HLA genes	Asian	GBD [73]	The alleles HLA-B*38:02, HLA-DRB1*16:02, HLA-DQA1*01:02, *HLA*-DQB1*05:02 and corresponding haplotypes might contribute to the development of GBD.
HLA genotyping [HLA class I alleles (24-A alleles and 48-B allele mix) and class II (24-DRB1 allele mix, *HLA-DRB3*, *HLA-DRB4*, *HLA-DRB5*, 11-DQA1 and 13-DQB1 allele mix)]	Polymerase chain reaction (PCR) with sequence specific primers (PCR-SSP)	Iranian	GBD [69]	HLA-A*68 and HLA-B*08 confer susceptibility to GBD, whereas HLA-A*33, HLA-DQB1*0201, and HLA-DQA1*0201 appear to have protective roles.
HLA genotyping (Class I genes, *HLA-A*, *HLA-B*, and *HLA-C*, as well as the Class II genes, *HLA-DRB1* and *HLA-DQB1. HLA-A*, *HLA-B*, and *HLA-C*)	NGS	Caucasians	GBD [70]	Significant associations between Grave’s ophthalmopathy (GO) and the HLA profile were demonstrated, with HLA-A*01:01, HLA-A*32:01, HLA-B*37:01, HLA-B*39:01, HLA-B*42:01, HLA-C*08:02, HLA-C*03:02, HLA-DRB1*03:01, HLA-DRB1*14:01, and HLA-DQB1*02:01 being genetic markers of increased risk of GO, and HLA-C*04:01, HLA-C*03:04, HLA-C*07:02, and HLA-DRB1*15:02 being protective alleles.
Three SNPs (rs3177928, rs7197, and rs3129878) of *HLA-DRA* genotypes	Multiplex PCR and high-throughput sequencing genotyping	Asian	GBD and HT [71]	rs3177928 and rs7197 correlated with AITD and GBD (compared with the healthy control group), but not with HT. However, rs3177928 and rs7197 correlated with AITD and HT in the allele model, dominant model, and overdominant model before and after gender and age adjustment, but not with HT.

Abbreviations: AITD: autoimmune thyroid disease, GBD: Graves-Basedow disease, HT: Hashimoto’s thyroiditis.

**Table 3:** Association of *PTPN22* C1858T SNPs with AITD susceptibility.

Association of *PTPN22* C1858T SNPs with AITD (Both, GBD, or HT)				
Population	Case/Controls	Genotype/Allele/SNPs	Association	References
Japanese	456/221	T-allele	No association	[79]
Japanese	334/179	C1858T	No association	[80]
Korean	212/225	T-allele	No association	[81]
Jordanian Arab	204/204	C1858T	No association	[82]
Polish	149/200	C1858T	No association	[83]
German	140/100	T-allele	Susceptible	[84]
Egyptian	60/60	C1858T	Susceptible	[85]
**Association of *PTPN22* C1858T SNPs only with GBD**				
Population	Case/controls	Genotype/allele/SNPs	Association	References
Mixed	3764/3328	C1858T	Susceptible	[86]
English	901/833	C1858T	Susceptible	[87]
English	768/768	C1858T	Susceptible	[88]
Polish	735/1216	C1858T	No association	[89]
English	549/429	C1858T	Susceptible	[90]
Polish	290/310	T-allele	Susceptible	[91]
Polish	166/154	C1858T	Susceptible	[92]
Indian Kashmiri	135/150	C1858T	No association	[93]
Latin-American	83/336	C1858T	Susceptible	[94]
Mixed	3 studies	T-allele	Susceptible	[95]

Abbreviations: AITD: autoimmune thyroid disease, GBD: Graves-Basedow disease, HT: Hashimoto’s thyroiditis, PTPN22: protein tyrosine phosphatase non-receptor type 22, SNPs: single nucleotide polymorphisms.

**Table 4:** Other candidate genes involved in AITD.

Gene	Functions	Association of SNPs with AITD	Populations	References
FCReceptor-like-3 (*FCRL3*)	Encode a number of proteins involved in the control of BL signaling	GBD	Asian and Caucasian descent	[198,199]
Secretoglobin 3A2 (*SCGB3A2*)	Encode uteroglobin related protein 1	HT	Croatian	[200]
*Interleukin-1B* and *Interleukin-4*	Encode cytokines, including some of the inflammatory type	GBD and HT	Tunisian, Iranian	[201,202]
*Interleukin-1 Receptor antagonist*	Modulate the autoimmune inflammatory response	GBD	English Caucasian, European	[203,204]
Tumor necrosis factor receptor (*TNFR*)	Modulate the autoimmune inflammatory response (involved in encoding the synthesis of proinflammatory cytokines)	AITD	Tunisians	[205]
*IFN-γ*	Works by activating cytotoxic TL, increasing cell-mediated cytotoxicity, and suppressing humoral immunity	HT	Japanese	[206]
AT-rich interactive domain (*ARID5B*)	Acts by inducing cell differentiation	GBD	Japanese	[207]
Neurexin 3 (*NRXN3*)	Is involved in cell recognition and cell adhesion	GBD	North American Caucasian	[208]
BL activating factor belonging to the TNF family (*BAAF*)	Plays an essential role in regulating the maturation, proliferation, differentiation, and survival of BL	GBD, AITD	Chinese, United Kingdom	[209,210,211]

Abbreviations: AITD: autoimmune thyroid disease, BL: B lymphocyte, GBD: Graves-Basedow disease, HT: Hashimoto’s thyroiditis, IFN: interferon, TL: T lymphocyte.

**Table 5:** Epigenetic studies and general findings in patients with AITD.

Endpoint	Disease	Findings	References
DNA methylation	GBD and AITD	Genome-wide screening revealed hypermethylated and hypomethylated genes, differentially methylated sites in TL CD4+ and CD8+ and impaired methylation, and increased expression of the ICAM1 gene	[222,226]
Histone tail modifications	GBD	Global reduction of histone 4 acetylation, reduction of histone 3 lysine 4 trimethylation (H3K4me3), and histone 3 lysine 27 acetylation (H3K27ac)	[223,227]
MicroRNA (miRNA) expression	GBD, HT, AITD	No expression of miR-154, miR-376b, and miR-431, increased levels of miR-22, miR-375, and miR-451; additionally, an altered expression of miRNAs with a resulting deregulated expression of messenger RNAs was found	[228,229]

Abbreviations: AITD: autoimmune thyroid disease, GBD: Graves-Basedow disease, HT: Hashimoto’s thyroiditis, TL: T lymphocyte.

**Table 6:** Microorganism, gut microbiota, and mechanism involved in AITD.

Microorganism	Most Important Mechanism	Other Mechanisms	References
*H. Pylori*	Molecular mimicry, cross-reactivity	*H. pylori* stimulates the proliferation of TL CD4+, which recognize *H. pylori* epitopes structurally similar to H/K/ATPase on the stomach parietal cells, the H/K/ATPase is also present on the thyroid gland, and the Th1 autoreactive on the thyroid gland induce apoptosis and synthesize proinflammatory cytokines	[234,235,236]
*Hepatitis C virus* (*HCV*)	Treatment with IFN-α	IFN-α can upregulate the expression of MHC class I, inducing the activation of cytotoxic TL, resulting in tissue damage and an inflammatory response; additionally, IFN-α can reduce the Treg function	[237,238]
*T. gondii*	Cross-reactivity of parasite antigens with antigens from the host	Molecular mimicry and the “bystander effect” via the activation of Toll-like receptors (TLR), thus inducing to the expansion of autoantibodies under aberrant conditions (for instance, excessive and/or chronic TLR activation)	[239,240]
*Human immunodeficiency virus* (*HIV*)	Immune reconstitution inflammatory syndrome	Molecular mimicry and direct cytopathic effect of HIV on the thyroid gland	[241]
*Herpes virus*	Viral superantigens (probably)	Bystander effects (probably)	[242,243]
*SARS-CoV-2*	Cross-reactivity	Molecular mimicry, cytokine storm	[244,245]
*Y. enterocolitica*	Cross-reactivity	Molecular mimicry	[246,247]
*B. burgdorferi*	Molecular mimicry	Cross-reactivity	[248]
*Hantavirus*	Viral superantigens	Molecular mimicry, activation of lymphocytes, cross-reactivity	[249,250]
*Saccharomyces*	Cross-reactivity	Bystander effect, intestinal permeability	[251,252]
*Gut microbiota*	Bacterial overgrowth, overactivation of the inflammasome, increased intestinal permeability	The short-chain fatty acids, metabolites of commensal bacteria fermentation of dietary fiber, could play a role in the development, functioning, and modulation of the immune system	[253,254]

Abbreviations: AITD: autoimmune thyroid disease, IFN: interferon, MHC: major histocompatibility complex, Th: T helper lymphocyte, TL: T lymphocyte, TNF: tumor necrosis factor, Treg: regulatory T lymphocyte.

**Table 7:** Nutritional factors related to AITD.

Nutritional Status	Molecular Mechanism	References
Iron deficiency	TPO undergoes post-translational modification as a consequence of iron deficiency, with the exposure of previously hidden epitopes or generating novel epitopes, which enhances the immunogenicity of TPO.	[256]
Iodine excess	Increased immunogenicity of highly iodinated Tg. Increase in the expression of intercellular adhesion molecule-1, on the thyrocyte. Increased in the production of thyroid-infiltrating Th cells, inhibition of Treg development, and an abnormal expression of TNF-related apoptosis (resulting in apoptosis and tissue destruction).	[257,258,259]
Selenium deficiency	Increased TL activation, while the T helper-1 (Th1)/T helper-2 (Th2) ratio may shift to a Th1-type response, increasing such cytokines as IL-2, TNF-α, IFN-γ, thereby reducing Treg and in parallel inhibiting an activation of DCs and macrophages by Toll-like receptor (TLR) cells.	[260,261,262]
Vitamin D deficiency	A lack of Vit-D causes a large number of BLs to proliferate and differentiate into plasma cells, which then secrete large amounts of IgG, IgE, and other immunoglobulins, causing thyroid cells to be damaged and triggering AITD. Vit-D is involved in the regulation of cytokines such as IL-1, IL-6, IL-17, TNF-, and leptin, which have been linked to AITD.	[263,264]
Gluten intake	The strongest association between gluten consumption and thyroid destruction appears to be based on a molecular mimicry mechanism between gut and thyroid tissue transglutaminase. In individuals with celiac disease, one of the autoantibodies produced is intestine tissue transglutaminase (which deaminates gliadin), and due to the presence of the transglutaminase in tissues other than intestinal, autoimmune cross-reactions with other tissues are possible. This enzyme is also found in the thyroid gland, and it was suggested that it is one of the reasons for celiac disease patients becoming more prone to HT.	[265,266]

Abbreviations: AITD: autoimmune thyroid disease, BL: B lymphocyte, DCs: dendritic cells, IFN: interferon, Ig: immunoglobulin, IL: interleukin, Th: T helper lymphocyte, TL: T lymphocyte, TNF: tumor necrosis factor, TPO: thyroid peroxidase, Treg: regulatory T lymphocyte, Vit-D: vitamin D.

**Table 8:** Non-genetic and nonnutritional factors most frequently associated with AITD.

Factors	Exposition	Findings	Probable Mechanisms	References
Endocrine disruptor	Mercury in consumed fish	Increased levels of TPOAbs and TgAbs	These compounds can inhibit the synthesis of thyroid hormones, either through a direct effect on thyroid iodine uptake (mediated by the NIS) or by altering the expression of genes involved in the synthesis of thyroid hormones or by altering the activity of thyroid hormones from TPO	[270]
Endocrine disruptor	High nitrate area	Higher frequency of TPOAbs positivity, higher frequency of thyroid hypoechogenicity, and higher TSH levels		[271]
Endocrine disruptor	Polychlorinated biphenyls; nitrate, thiocyanate, and soy isoflavone	Higher frequency of thyroid antibodies		[272,273]
Endocrine disruptor	Organochlorine pesticides, polychlorinated biphenyls	Higher frequency of thyroid antibodies		[274,275,276]
Endocrine disruptor	Vanadium pentoxide	There was an increased secretion of chemokines CXCL8, CXCL9, CXCL10, and CXCL11 from thyrocytes		[277,278,279]
Smoking	Smoking exposition	Smoking increases the risk of GBD ophthalmopathy beyond the risk associated with GBD alone. Smoking cessation may lead to a decrease in morbidity from GBD, especially in women. Additionally, both TPOAbs-positive and TRAbs-positive individuals were exposed to a higher amount of smoking pack-years	Thiocyanate can inhibit the transport and metabolism of iodine, and therefore the synthesis of thyroid hormones	[280,281,282]
Alcohol	Alcohol intake	Alcohol consumption seems to confer protection against the development of overt autoimmune hypothyroidism and GBD	Loss of TL NK activity, or due to changes in the immunoglobulin level, an alteration in the production of cytokines is also described and, finally, a probable effect mediated by the modification of the intestinal microbiota	[283,284,285,286]
Stress	Stress exposition	Stress can be one of the environmental factors for thyroid autoimmunity. Anxiety is positively associated with elevated TSH and TgAbs levels	It has been postulated that stress acts as an immune-modulator by directly or indirectly affecting the immune system via the nervous and endocrine systems, thereby triggering AITD in susceptible individuals	[287,288,289]
Drugs	Amiodarone, lithium, IFN-γ	Drugs containing iodine may induce or exacerbate AITD in susceptible individuals; lithium induces goiter and hypothyroidism (20%) and increases the risk of AITD in susceptible individuals	Increased iodine intake (amiodarone) increases Tg antigenicity.IFN-γ inducible CXC chemokines may play an important part in triggering AITD in susceptible patients	[290,291,292,293]

Abbreviations: AITD: autoimmune thyroid disease, GBD: Graves-Basedow disease, IFN: interferon, NIS: Na+/I^−^ symporter, NK: natural killer, TL: T lymphocyte, Tg: thyroglobulin, TgAbs: Tg autoantibodies, TPO: thyroid peroxidase, TPOAbs: TPO autoantibodies, TRAbs: thyroid stimulating hormone receptor autoantibodies.

**Table 9:** Main features of thyroid antigens.

Features	Tg	TSHR	TPO	Pendrin	NIS
Protein	Iodinated glycoprotein	G protein-coupled receptor	Hemoprotein enzyme	Hydrophobic transmembrane glycoprotein	Membrane glycoprotein
Amino acids	2748	743	933	780	643
Molecular weight (kDa)	660	85	105–110	86	70–90
Epitope localization	Predominantly central region and C-terminus	Predominantly A-subunit	Predominantly myeloperoxidase-like domain and, to a lesser extent, complement control protein-like domain	Apical membrane of thyrocytes	Predominantly extramembranous regions
Function	Iodide storage and thyroid hormonogenesis	Activate signaling pathways that promote the expression of genes that control thyrocyte growth and differentiation, and the synthesis of thyroid hormones	Thyroid hormone biosynthesis	Iodide transport in thyroid cells; pendrin mediates electroneutral exchange of chloride for bicarbonate across the plasma membrane of epithelial cells	Cellular uptake of iodide
Chromosomal location	8q24	14q31	2p25	7q22-31	19p12-13.2

Abbreviations: NIS: Na+/I- symporter, Tg: thyroglobulin, TPO: thyroid peroxidase, TSHR: thyroid stimulating hormone receptor.

## Data Availability

Not applicable.
